# A New Poison

**Published:** 1857-08

**Authors:** 


*A New Poison.*—M. de Luca has just communicated to the Academy of Sciences the discovery of the poisonous principle of the cyclamen Europæum, or common sowbread. This tuberculous plant has long been used in medicine as a violent purgative, and externally as a resolvent and a remedy for the earache, but it was not known that it contained a powerful poison, producing effects not unlike those of the curara, which the Indians of the Rio Negro use to poison their arrows with. M. de Luca obtains it by digesting the root for 45 days in alcohol, then pounding the root, digesting it again in a fresh quantity of alcohol, and repeating this process until the pulp had lost its acrid taste. All the tinctures thus obtained are then left to a spontaneous evaporation in a cellar. At the end of about forty days a whitish substance is deposited, which, after being repeatedly washed in boiling alcohol, is left to dry in the dark. The cyclamine, or vegetable base of the cyclamen thus produced, is white, opaque and brittle, and emits no particular smell; it absorbs the humidity of the air, becomes transparent and gelatinous in water, and assumes a dark color when exposed to the action of light. It is a curious fact that, while pigs can eat any quantity of the root with impunity, not only the active principle itself, but even the natural juice of the root, acts as a poison on small fish, if mixed with the water in which they are, in the proportion of 1 to 3000. Four grammes of the juice injected into the trachea of a rabbit caused it to die in convulsions in ten minutes. Bromine appears to be an antidote to this poison, or at least to mitigate its effects considerably; it has the same neutralizing power over the curara poison. [Med. Times & Gaz.

